# Cardiac copper content and its relationship with heart physiology: Insights based on quantitative genetic and functional analyses using BXD family mice

**DOI:** 10.3389/fcvm.2023.1089963

**Published:** 2023-02-02

**Authors:** Akhilesh Kumar Bajpai, Qingqing Gu, Buyan-Ochir Orgil, Fuyi Xu, Carolina Torres-Rojas, Wenyuan Zhao, Chen Chen, Athena Starlard-Davenport, Byron Jones, Djamel Lebeche, Jeffrey A. Towbin, Enkhsaikhan Purevjav, Lu Lu, Wenjing Zhang

**Affiliations:** ^1^Department of Genetics, Genomics and Informatics, The University of Tennessee Health Science Center, Memphis, TN, United States; ^2^Department of Cardiology, Affiliated Hospital of Nantong University, Nantong, Jiangsu, China; ^3^Department of Pediatrics, The University of Tennessee Health Science Center, Memphis, TN, United States; ^4^Le Bonheur Children’s Hospital, Children’s Foundation Research Institute, Memphis, TN, United States; ^5^School of Pharmacy, Shandong Technology Innovation Center of Molecular Targeting and Intelligent Diagnosis and Treatment, Binzhou Medical University, Yantai, Shandong, China; ^6^Department of Physiology, College of Medicine, The University of Tennessee Health Science Center, Memphis, TN, United States; ^7^Pediatric Cardiology, St. Jude Children’s Research Hospital, Memphis, TN, United States

**Keywords:** BXD, cardiac Cu homeostasis, systems genetics, hypertrophy, cardiomyopathy, genetic mapping, quantitative trait loci, gene expression

## Abstract

**Background:**

Copper (Cu) is essential for the functioning of various enzymes involved in important cellular and physiological processes. Although critical for normal cardiac function, excessive accumulation, or deficiency of Cu in the myocardium is detrimental to the heart. Fluctuations in cardiac Cu content have been shown to cause cardiac pathologies and imbalance in systemic Cu metabolism. However, the genetic basis underlying cardiac Cu levels and their effects on heart traits remain to be understood. Representing the largest murine genetic reference population, BXD strains have been widely used to explore genotype-phenotype associations and identify quantitative trait loci (QTL) and candidate genes.

**Methods:**

Cardiac Cu concentration and heart function in BXD strains were measured, followed by QTL mapping. The candidate genes modulating Cu homeostasis in mice hearts were identified using a multi-criteria scoring/filtering approach.

**Results:**

Significant correlations were identified between cardiac Cu concentration and left ventricular (LV) internal diameter and volumes at end-diastole and end-systole, demonstrating that the BXDs with higher cardiac Cu levels have larger LV chamber. Conversely, cardiac Cu levels negatively correlated with LV posterior wall thickness, suggesting that lower Cu concentration in the heart is associated with LV hypertrophy. Genetic mapping identified six QTLs containing a total of 217 genes, which were further narrowed down to 21 genes that showed a significant association with cardiac Cu content in mice. Among those, *Prex1* and *Irx3* are the strongest candidates involved in cardiac Cu modulation.

**Conclusion:**

Cardiac Cu level is significantly correlated with heart chamber size and hypertrophy phenotypes in BXD mice, while being regulated by multiple genes in several QTLs. *Prex1* and *Irx3* may be involved in modulating Cu metabolism and its downstream effects and warrant further experimental and functional validations.

## Introduction

Copper (Cu), a trace element, is essential for many Cu-binding enzymes and proteins that are required for various physiological functions, including regulation of mitochondrial respiration, iron metabolism, antioxidant defense, and maintenance of the extracellular matrix (ECM) in mammals (1, 2). A few examples of such enzymes and proteins (chaperones) are cytochrome c oxidase (CCO), superoxide dismutase (SOD), metallothionein (MT), ceruloplasmin (CP), dopamine-beta-hydroxylase (DBH), and lysyl oxidase. Several other Cu-chaperones deliver Cu to CCO; these include CCO copper chaperone 11 (COX11), COX17, COX19, COX23, and synthesis of CCO1 (SCO1) and SCO2. They are essential for oxidative phosphorylation in the heart. Furthermore, Cu has been shown to regulate target gene selectivity of hypoxia-inducible factor 1 (HIF-1), a transcription factor that regulates cellular responses to hypoxia, by affecting its binding to the gene promoters (3). Importantly, normal Cu homeostasis depends on a very narrow range of Cu concentrations in tissues and fluids, and therefore, Cu-uptake and -utilization processes are tightly regulated by coordinated functions of several transporter proteins and enzymes (4). Copper transporter 1 (CTR1) is the evolutionarily conserved high-affinity Cu transporter, while another Cu transporter, divalent metal transporter 1 (DMT1), has been shown to compensate for Cu uptake in the absence of CTR1 (5). The Cu efflux from cells is mainly regulated by ATPases, such as ATP7A and ATP7B. Mutations in ATP7A and ATP7B genes cause inherited disorders, known as Menkes and Wilson diseases, respectively (2). While ATP7A mutations cause Cu deficiency resulting in developmental delay, hypotonia, and nervous system deterioration, mutations in ATP7B cause Wilson’s disease that results in excessive Cu accumulation, mainly in the liver and brain.

Cu content in the body is an important parameter for physiological functions in humans, and imbalanced Cu concentrations have been shown to associate with heart diseases. Wilson’s disease patients with Cu overload exhibit a higher risk of atrial fibrillation with sudden cardiac death and heart failure (6, 7). The marginal dietary Cu restriction demonstrated a potentially adverse effect on the cardiovascular system (8), while dietary Cu deficiency caused structural and functional pathologies in the heart, including cardiac hypertrophy with deposition of lipid droplets and excessive collagen accumulation, disruptive cristae, vacuolization of mitochondria, and increased mitochondrial biogenesis (9). Supplementation by a Cu-adequate diet has been shown to reverse cardiac hypertrophy in subjects with dietary Cu deficiency (10) and in patients with hypertrophic cardiomyopathy (HCM) carrying mutations in Cu chaperones, SCO1 and SCO2 (11). Moreover, Cu repletion reversed the HCM phenotype in Cu-deficient and pressure overload animal models *via* SOD-mediated HIF-1 activation of VEGF (vascular endothelial growth factor) expression and angiogenesis (10–13). Disturbed Cu metabolism has also been connected to ischemic heart disease and heart failure involving dysfunction of CP, SOD1, and SOD3 chaperones (11). Mice with a heart-specific knockout of CTR1 (hCtr1^–/–^) suffered from cardiac Cu deficiency that resulted in severe cardiomyopathy (14). Remarkably, these hCtr1^–/–^ mice exhibited increased serum Cu levels and a concomitant decrease in hepatic Cu storage capacity *via* ATP7A mobilization in the liver. While fluctuations in cardiac Cu content have been shown to cause cardiac pathologies, the regulation and genetic basis that influence cardiac Cu content, and whether this is associated with the physiological parameters of heart morphology and function remain to be understood.

The goal of this study was to identify the genes and chromosomal regions that influence Cu concentrations in the heart and correlate cardiac Cu content with heart structure and function in a murine genetic reference population (GRP) of the recombinant-inbred (RI) BXD family. The BXD family has been bred specifically for systems genetics studies of complex traits to identify causal and modifier candidate genes and their phenotype-modulating effects on a whole genome level. The BXD family consists of over 100 highly diverse lines of mice that descend from two independent advanced RI crosses between C57BL/6J (B6) and DBA/2J (D2) parental strains (15–17). The father of the BXDs (D2 strain) is an inherent model of HCM, whereas the mother of BXDs (B6 strain) has normal cardiac morphology and function (18). The phenotype and severity of HCM greatly vary among patients with the same causal gene mutations as well as among BXD strains, and these variations in an individual’s risk are regulated by genetic background, metabolic features, and various environmental factors (19, 20). In this study, we hypothesized that Cu content is associated with cardiac function, thus we measured Cu concentrations in the myocardium and collected echocardiographic data from multiple BXD strains and their parental lines. Furthermore, quantitative trait loci (QTL) analysis was performed using GeneNetwork (GN),^1^ which identified six QTLs and 21 possible candidate genes that are associated with cardiac Cu metabolism and cardiac function.

## Materials and methods

### Animals and tissue harvesting

All animals were housed in an environmentally controlled animal facility (12:12 light/dark cycle) with free access to water and standard chow diet, standard rodent diet 7,912 (irradiated Teklad LM485, Envigo) containing 240 ppm Fe, 23 ppm Cu, and 63 ppm Zn throughout their life or until sacrifice. The tap water contained 1.6, 0.5, and 4.3 ppm Fe, Cu, and Zn, respectively. All experimental protocols were performed in accordance with the NIH Animal Care guidelines and were approved by The University of Tennessee Health Science Center Animal Care and Use Committee (approval code # 21-0310).

A total of 313 mice from 72 BXD RI strains were used for Cu content measurement (mean *n* > 4/strain) and 162 mice from 73 BXD strains plus their four parental strains, C57BL/6J (B6), DBA/2J (D2), B6D2F1 and D2B6F1, were used for the generation of heart gene expression data (mean *n* > 2/strain). Animals were euthanized under isoflurane anesthesia. The heart was dissected, and the atria, aorta, and surrounding tissues were removed. The ventricles were washed in phosphate-buffered saline and frozen in isopentane with dry ice for further measurement of Cu content and generation of gene expression data.

### Measurement of Cu content

The heart tissue was wet-washed with nitric acid and analyzed by total reflection X-ray fluorescence spectroscopy using an S2 PICOFOX instrument (Bruker, Berlin). The samples were measured for 500 s and the values for Cu were normalized to tissue wet weights. Metal concentration (μg/g) is reported as mean ± standard error of the mean (SEM).

### Measurement of echocardiographic parameters

Transthoracic two-dimensional and Doppler echocardiography was used to evaluate heart function and morphology in BXD mice. Experiments were conducted in BXD, B6, and D2 strains (44 strains) at 4–6 months of age (*n* > 5/strain). The chest of the mice was treated with a chemical hair remover day prior. Mice were anesthetized by oxygenated 1–2% isoflurane, and core temperature and heart rate were maintained using a heated platform set at 37^°^C, while echocardiography was performed using a Vevo2100 Micro-Imaging System (VisualSonics Inc., Toronto, Canada). Fractional shortening (FS%), ejection fraction (EF%), left ventricular volumes end-diastolic and end-systolic (LVVol;d and LVVol;s, respectively), LV internal diameters end-diastolic and end-systolic (LVID;d and LVID;s, respectively), interventricular septum end-diastolic and end-systolic (IVS;d and IVS;s, respectively) and LV posterior wall thickness end-diastolic and end-systolic (LVPW;d and LVPW;s, respectively), stroke volume and cardiac output (CO) were calculated as reported previously (19).

### QTL mapping

The QTL mapping for identifying genetic loci associated with Cu concentration in mouse hearts was performed using Genome-wide efficient mixed-model association (GEMMA)^2^ method in GN (see text footnote 1) (21). It maps traits with correction for kinship among samples using a linear mixed model method and incorporates the Leave One Chromosome Out (LOCO) method to ensure that the correction for kinship does not remove useful genetic variance near each marker. Further, the markers were filtered to include only those with minor allele frequencies above the default threshold of 0.05. The suggestive and significant thresholds used for a genome-wide scan were –log(p) of 3.0 and 4.0, respectively. The threshold is based on one unit of –log(p) being equivalent to one unit of the logarithm of the odds (LOD) value, where LOD = likelihood ratio statistic (LRS)/4.61. A total of 21,054 informative single nucleotide polymorphism (SNP) genotype markers were used for the analysis. The BXD genotype file can be accessed and reviewed on GN. A 1.5-LOD confidence interval was used for identifying the candidate genes.

### Cardiac gene expression data

The gene expression data, “NHLBI BXD All Ages Heart RNA-Seq (Nov20) TMP Log2” used in the current study was generated from hearts of 73 BXD strains plus their parental strains—C57BL/6J (B6), DBA/2J (D2), B6D2F1 and D2B6F1 (one male and one female per strain for most of the strains) in our laboratory recently. This data set can be accessed at our GN website (see text footnote 1) with the accession number GN1028 (22).

### RNA isolation and sequencing

Briefly, total RNA was extracted using miRNeasy Mini Kit (Qiagen, Hilden, Germany) according to the manufacturer’s instructions. Approximately, 30 mg of left ventricle tissue was added into a 2 mL tube containing 700 μL QIAzol Lysis Reagent and one 5 mm stainless steel bead (Qiagen, Hilden, Germany). The tissue was homogenized in a Tissue Lyser II (Qiagen, Hilden, Germany) for 2 min with a speed frequency of 30 r followed by incubation for 5 min. Then, 140 μL chloroform was added into the homogenate, shaken vigorously for 15 s, and centrifuged at 12,000 × g for 15 min at 4^°^C. Following centrifugation, 280 μL upper aqueous was transferred into a new collection tube containing 500 μL 100% ethanol. The mixture was loaded into a RNeasy Mini Kit spin column (Qiagen, Valencia, CA, USA), and then washed once with RNeasy wash buffer (RWT) and twice with RNeasy wash buffer with ethanol (RPE) for RNA elution. All RNA samples were analyzed using an Agilent 2100 Bioanalyzer (Agilent Technologies, Santa Clara, CA, USA), and samples with optical density 260/280 > 1.8 and RNA integrity number > 8.0 were used for library preparation.

One microgram of RNA was used for complementary DNA (cDNA) library preparation using a NEBNext^®^ Ultra RNA Library Prep Kit for Illumina^®^ (cat# E7420S, New England Biolabs, Ipswich, MA, USA) according to the manufacturer’s protocol. Briefly, mRNA was enriched using oligo(dT) beads followed by two rounds of purification and fragmented randomly by adding a fragmentation buffer. The first strand cDNA was synthesized using random hexamer primers, after which a custom second-strand synthesis buffer (Illumina, San Diego, CA, USA), dNTPs, RNase H, and DNA polymerase I were added to generate the second strand (double-stranded cDNA). After a series of terminal repairs, poly-adenylation, and sequencing adaptor ligation, the double-stranded cDNA library was completed following size selection and polymerase chain reaction (PCR) enrichment. The resulting 250–350 bp insert libraries were quantified using a Qubit 2.0 fluorometer (Thermo Fisher Scientific, Waltham, MA, USA) and quantitative PCR. Size distribution was analyzed using an Agilent 2100 Bioanalyzer (Agilent Technologies, Santa Clara, CA, USA). The qualified libraries were sequenced on an Illumina NovaSeq Platform (Illumina, San Diego, CA, USA) using a paired-end 150 run (2 × 150 bases). On average, 40 million raw reads were generated from each library.

### Alignment and quantification of RNA sequencing data

For mapping of the RNA-seq reads, mouse reference genome (GRCm38) and gene model annotation files were downloaded from the Ensembl genome browser (23).^3^ The reference genome was then indexed and paired-end reads were aligned to the indices using STAR v2.5.0a (24). The algorithm employed in STAR uses sequential maximum mappable seed search in uncompressed suffix arrays followed by seed clustering and stitching procedure, which can generate a precise mapping result for the junction reads. FeatureCount v0.6.1 was used to count the number of read(s) mapped to each gene (25). Transcripts Per Million (TPM) was calculated for each gene based on the length of the gene and the number of reads mapped to that gene. In this normalization, the sum of all TPMs (gene level) is equal to 1,000,000. The TPM values were further rescaled to log2 (TPM + 1).

### Genetic correlation analysis

Genetic correlation analysis was performed on our GN portal using the Pearson correlation coefficient to identify gene-phenotype and gene-gene associations. For gene-phenotype correlation analysis, the Cu concentration phenotype (GN phenotype ID: 21404) was correlated with the mRNA levels of genes in “The NHLBI BXD All Ages Heart RNA-Seq (Nov20) TMP Log2” dataset.

### Identification of candidate genes

A 1.5-LOD confidence interval was used for identifying potential candidate genes associated with the effect of Cu concentration in BXD mice hearts. We employed a scoring system (scores ranging from 0 to 10) to prioritize the candidate genes. The scoring system contains five different parameters, and each parameter was assigned a different score based on its overall significance. The reason for selecting these five parameters was to finally shortlist gene(s) that are important for the studied phenotype based on their tissue expression, significant association with the phenotype being studied, genotype as well as their known/studied functions in the context of the phenotype.

#### Expression in the heart (1 score)

Expression of genes at a particular level in a specific tissue is an important requirement for their functions to be carried out. Thus, genes having a higher expression in the heart tissue were prioritized, ensuring the presence of enough number of mRNA copies to carry out the functions. Hence, genes with a mean expression of ≥ 2 were assigned a score of 1.

#### Coding DNA variants (2 score)

The location of the variants in the gene sequence can affect the protein sequence and function. The variants, such as non-synonymous, frameshift, stop gain, or loss are particularly vital in affecting the gene function. Hence, using our previously generated genetic variant calls by whole genome resequencing (26) of the two parental strains, B6 and D2, the coding sequence polymorphisms for genes located in the QTL interval were evaluated. If a gene harbored a coding sequence variant, then a score of 2 was assigned.

#### Gene-phenotype correlation (2 score)

A significant gene-phenotype correlation is an important criterion that indicates the importance of a gene in a particular phenotype. Hence, only those genes that had a significant correlation with Cu concentration (*P* < 0.05) were selected and assigned a score of 2. The gene-phenotype correlation was done by Pearson correlation coefficient analysis in GN (see text footnote 1) (22).

#### Cis-regulation (2 score)

For the genes located in the 1.5-LOD QTL interval, expression QTL (eQTL) mapping was performed. A score of 2 was assigned for the *cis*-regulated genes. A gene was *cis*-regulated if the eQTL is located within 5 Mb flanking regions of this gene with a “maximum LRS” > 14.

#### Functional relevance (3 score)

To determine if the QTL genes are functionally important in cardiac-related phenotypes, the following public databases/resources were used: Mouse Genome Informatics (MGI)^4^ (27), Rat Genome Database (RGD)^5^ (28), International Mouse Phenotyping Consortium (IMPC)^6^ (29) and Genome-Wide Association Study (GWAS) Catalog (30).^7^ These databases were queried with cardiac or cardiovascular disease-related keywords, such as “heart,” “cardiac,” “cardiomyopathy,” “cardiomyocyte proliferation,” “angiogenesis,” “myocardial,” “myocardial ischemia,” “cardiovascular system disease,” or “heart disease” to obtain the associated genes. The phenotypes obtained from the GWAS catalog or IPMC databases were significant with a *P*-value < 1 × 10^–4^. In addition, genes related to the “Cu transport” from Gene Ontology (GO)^8^ (31) and MGI databases, and those related to “Cu homeostasis” pathway from WikiPathways database^9^ (32) were obtained. As Cu is known to regulate target gene selectivity *via* HIF-1 binding to the gene promoters (3), genes related to mouse “HIF-1 signaling pathway” from Kyoto Encyclopedia of Genes and Genomes (KEGG)^10^ database (33) were retrieved.

The genes obtained from these resources were matched to the QTL genes, and if a potential candidate gene was implicated in any of the above phenotypes, processes, or pathways, then a score of 3 was assigned. The scores across all five parameters were summed for each gene and genes with a total score of ≥ 6 (higher than the median score of 5) were selected as candidate genes for further analysis.

## Results

### Genomic loci associated with cardiac Cu concentration in BXD mice

**FIGURE 1:**
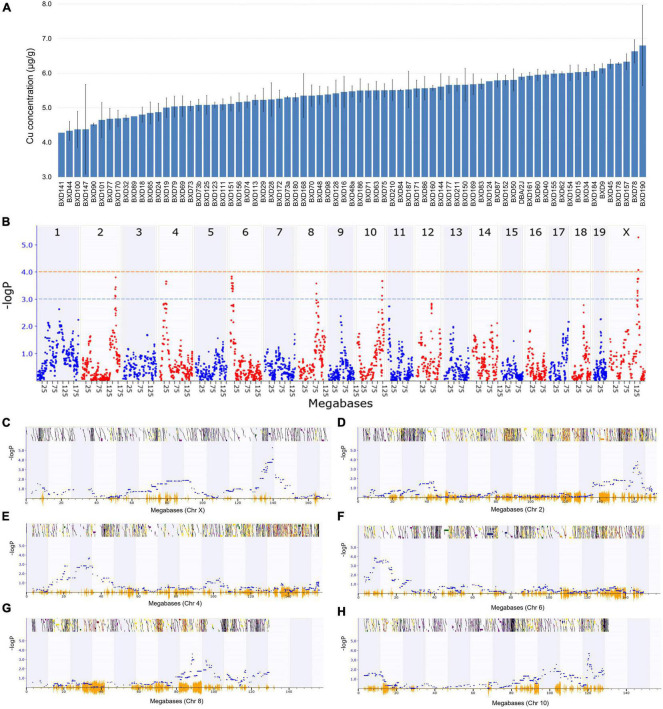
Genetic mapping of the cardiac copper (Cu) concentration in BXD family mice. **(A)** Bar plot showing Cu concentration in the myocardium of BXD mice. The x-axis shows the BXD and the parental strains, and the y-axis shows Cu levels in μg/g. **(B)** Manhattan plot indicating the genomic loci correlated with Cu concentration across all mouse chromosomes. The x-axis indicates the genomic position, while the y-axis shows the –log(P), a measurement of the linkage between Cu level and genomic region. The blue and red dotted lines indicate suggestive (–logP of 3) and significant (–logP of 4) genome-wide thresholds, respectively. **(C–H)** Genomic loci correlated with Cu concentration either with a significant or suggestive threshold in specific chromosomes. The x-axis indicates the genomic position, and y-axis denotes the –log(P) value.

Among the BXD strains, BXD190 had the highest (6.8 ± 1.2 μg/g) and BXD141 had the lowest (4.3 μg/g) Cu concentrations (Figure 1A). The parental strain, DBA/2J had a Cu level of 5.9 ± 0.1 μg/g, which was higher than the average Cu level (5.4 ± 0.1 μg/g) across all BXD strains. GEMMA mapping was performed to identify genetic loci associated with cardiac Cu concentration in BXD mice. As shown in Figures 1B, C, we identified a significant QTL on chromosome (Chr) X at 139 ∼ 141 Mb (peak LRS = 24.26), where one unit of LOD is equivalent to 4.61 LRS. Furthermore, with a threshold of 3 LOD (LRS = 13.8), five suggestive QTLs were mapped on different chromosomes (Figures 1D–H), as follows: Chr 2 at 159.4 ∼ 161.9 Mb (peak LRS = 17.43), Chr 4 at 29.2 ∼ 34 Mb (peak LRS = 16.74), Chr 6 at 8.4 ∼ 15.8 Mb (peak LRS = 17.54), Chr 8 at 88 ∼ 90 Mb (peak LRS = 16.4), and Chr 10 at 119.5 ∼ 120.7 Mb (peak LRS = 16.78).

### Candidate genes modulating cardiac Cu concentration in BXD mice

For prioritizing the candidates, genes within the 1.5-LOD confidence interval were selected. To further narrow down the candidate genes, we employed a comprehensive strategy, which included five different parameters, such as *cis*-regulation, coding sequence variants, expression in heart tissue, significant correlation with the phenotype(s), and functional relevance of the genes. The data associated with the first four parameters were collected from our GN portal, whereas the functional association of the genes was retrieved from various publicly available resources, such as MGI, IPMC, RGD, KEGG pathway, WikiPathways, and GO. Based on these data, the genes were assigned a score from 0 to 10, and those attaining a score of at least 6 (higher than the median score threshold of 5) were considered candidates for further analysis. A score of six also ensures that the selected genes qualify majority of the considered parameters.

**FIGURE 2:**
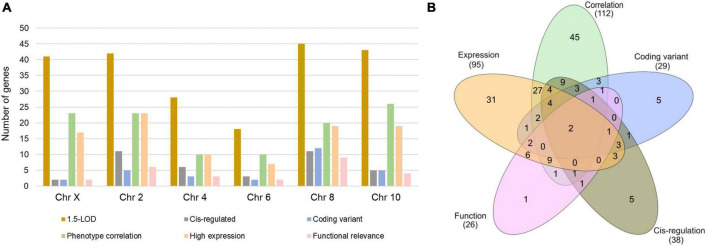
Number of genes within 1.5-LOD interval across the significant and suggestive QTLs. **(A)** Number of genes in each category for each indicated chromosome. **(B)** Number of exclusive and shared genes across five selection criteria.

The 1.5-LOD region across six QTLs contained a total of 217 genes with 41 from Chr X, 42 from Chr 2, 28 from Chr 4, 18 from Chr 6, 45 from Chr 8, and 43 from Chr 10 (Figure 2A). Interestingly, 12 of the 45 Chr 8 genes had coding sequence variants, whereas this number was comparatively much lower for other chromosomes. Approximately 60% of the Chr 10 genes within 1.5-LOD were significantly correlated with Cu phenotype. Functional relevance was lowest for Chr X genes, while it was highest for Chr 8 genes. Furthermore, approximately 25% of the Chr 2 and Chr 8 genes were *cis*-regulated (Figure 2A). Among the 217 genes, 112 were significantly correlated with Cu-concentration phenotype (GN phenotype ID: 21404), 29 had coding sequence variants, 38 were *cis*-regulated and 95 genes were expressed with a mean TPM value of ≥ 2. Furthermore, 26 of the genes within the 1.5-LOD region were functionally relevant being involved in one of the cardiac phenotypes or associated with Cu-related pathways or biological processes. Two genes (*Prex1* and *Irx3*) were common to all five categories (Figure 2B).

Finally, after scoring the 1.5-LOD interval genes based on different parameters, 21 genes across six QTLs (Chr X, 2, 4, 6, 8, and 10) with a score of ≥ 6 were selected as candidate genes that may have an important effect in modulating Cu concentration in the murine heart (Table 1). Each of the Chr 2 and Chr 8 contributed six genes to the candidate list. There were three genes each from Chr 4 and 10, two genes from Chr 6, and one gene from Chr X. The top two genes from Chr 2 were *Prex1* (phosphatidylinositol-3,4,5-trisphosphate-dependent Rac exchange factor 1) and *Fam83d* (family with sequence similarity 83, member D) with a score of 10 and 9, respectively. The top two genes from Chr 8 were *Irx3* (Iroquois-related homeobox 3) and *Usb1* (U6 snRNA biogenesis 1) which had a score of 10 and 7, respectively. Interestingly, *Prex1* (Chr 2: 166.566342) and *Irx3* (Chr 8: 91.798525) attained the maximum score of 10 (Table 1). Furthermore, *Fam83d* (Chr 2: 158.768093) was the next best candidate with a score of 9. A few of the candidate genes, such as *Slc35a1* (solute carrier family 35 member 1) encoding CMP-sialic acid transporter, *Ica1* (islet cell autoantigen 1), *Usb1*, and *Slc26a10* from chromosome 4, 6, 8, and 10, respectively, did not have any score for functional relevance. However, they received a full score for the remaining four categories and attained a score of 7, suggesting that these genes may be novel candidates for Cu phenotype and further studies are required for clarification.

**TABLE 1:** Candidate genes associated with cardiac Cu phenotype identified in significant and suggestive QTLs.

Gene symbol	Gene ID	Chr	Scores assigned for different categories					
			**Mean expression**	**Coding variant**	***Cis*-regulation**	**Correlation**	**Function**	**Total**
*Armcx1*	78248	X	1	0	0	2	3	6
* **Fam83d** *	**71878**	**2**	**0**	**2**	**2**	**2**	**3**	**9**
*Ift52*	245866	2	1	0	0	2	3	6
*Ada*	11486	2	1	0	0	2	3	6
*Matn4*	17183	2	0	2	2	2	0	6
*Ctsa*	19025	2	1	0	0	2	3	6
* **Prex1** *	**277360**	**2**	**1**	**2**	**2**	**2**	**3**	**10**
*Ankrd6*	140577	4	0	0	2	2	3	7
*Slc35a1*	24060	4	1	2	2	2	0	7
*C9orf72*	73205	4	1	0	0	2	3	6
*Col28a1*	213945	6	0	2	2	2	0	6
*Ica1*	15893	6	1	2	2	2	0	7
* **Irx3** *	**16373**	**8**	**1**	**2**	**2**	**2**	**3**	**10**
*Mmp2*	17390	8	1	0	0	2	3	6
*Gnao1*	14681	8	1	0	0	2	3	6
*Usb1*	101985	8	1	2	2	2	0	7
*Csnk2a2*	13000	8	1	0	0	2	3	6
*Gins3*	78833	8	0	2	2	2	0	6
*Cand1*	71902	10	1	0	0	2	3	6
*Lrig3*	320398	10	0	2	0	2	3	7
*Slc26a10*	216441	10	1	2	2	2	0	7

Important candidate genes based on overall scoring are underlined and highlighted in **bold**.

### Association between cardiac Cu concentration and cardiac phenotypes in BXD mice

**FIGURE 3:**
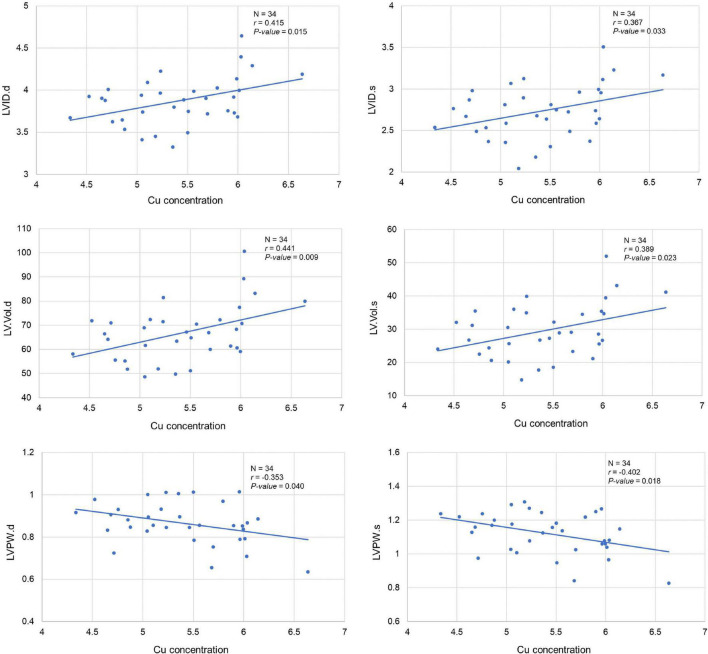
Correlation between cardiac copper (Cu) concentration and echo phenotypes (LVID, LV.Vol, and LVPW). The x-axis indicates the Cu concentration (μg/g), and y axis denotes various echocardiography parameters. The Pearson correlation coefficient was used to determine the relationship between cardiac Cu concentration and echo phenotypes. The Pearson correlation *r* and *P*-values are indicated in the plots. LV, left ventricular; s, end-systole; d, end-diastole; LVID, LV internal diameter; LV.Vol, LV volume; LVPW, LV posterior wall.

**FIGURE 4:**
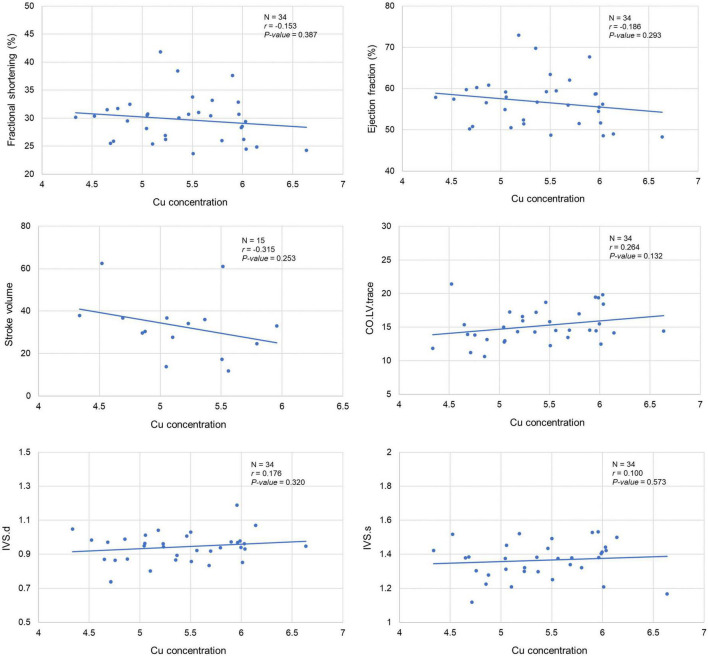
Correlation between cardiac copper (Cu) concentration and echo phenotypes (FS%, EF%, stroke volume, CO, and IVS). The x-axis indicates the Cu concentration (μg/g), and y axis denotes the echocardiography parameters. The Pearson correlation coefficient was used to determine the relationship between cardiac Cu concentration and echo phenotypes. The Pearson correlation *r* and *P*-values are indicated in the plots. LV, left ventricular; s, end-systole; d, end-diastole; CO, cardiac output; IVS, interventricular septum.

Our analysis clearly showed a significant correlation between cardiac Cu concentration and various echocardiographic phenotypes. For instance, LVID at end-diastole (*r* = 0.415; *p* = 0.015) and end-systole (*r* = 0.367; *p* = 0.033) were significantly positively correlated with cardiac Cu levels (Figure 3). Similarly, a positive correlation was also observed between Cu concentration and LV volumes at end-diastole (*r* = 0.441; *p* = 0.009) and end-systole (*r* = 0.389; *p* = 0.023) (Figure 3). In addition, LVPW thickness at end-diastole and end-systole significantly negatively correlated with Cu concentration (*r* = -0.353; *p* = 0.040 and *r* = -0.402; *p* = 0.018, respectively). Furthermore, other echocardiographic phenotypes, including FS%, EF%, stroke volume, CO, and IVS although correlated with Cu concentration, did not attain statistical significance. While a negative correlation was observed for FS% (*r* = -0.153; *p* = 0.387), EF% (*r* = -0.186; *p* = 0.293), and stroke volume (*r* = -0.315; *p* = 0.253), a positive correlation was noted for CO (*r* = 0.264; *p* = 0.132), IVS.d (*r* = 0.176; *p* = 0.320) and IVS.s (*r* = 0.100; *p* = 0.573) with Cu concentration (Figure 4). The values recorded for the echocardiographic parameters and Cu phenotype are provided in Supplementary File 1.

## Discussion

The strictly balanced concentrations of Cu in peripheral organs and in circulation are crucial for various biological processes in mammals, and imbalances in Cu concentrations affect multiple tissues and organs resulting in a broad range of disorders. While dietary Cu deficiency results in HCM, heart ischemia, and heart failure (9), Cu excess is also toxic and causes cardiomyopathy with lethal arrhythmias and heart failure in patients with Wilson’s disease (7). Notably, Cu content in the heart has been shown to regulate the systemic Cu homeostasis and the Cu content in the liver, the main organ that is responsible for excreting excess Cu from the body (4, 14). However, major questions persist about the association of cardiac Cu concentrations with heart morphology and function and its underlying genetic regulation. To this end, we have previously applied a systems genetics approach using the BXD family of RI mice, a murine GRP that has previously been successfully used for several QTL analyses to identify candidate genes and gene networks that underlie the effects of trace metals in the brain (34, 35). Similarly, in the current study, we showed that the cardiac Cu concentration in BXD mice is significantly correlated with various echocardiographic parameters. Our results demonstrate that BXD strains with larger LV internal diameters and volumes have higher Cu content in the myocardium, indicating that excessive Cu accumulation in the heart is related to LV dilation. Supporting this correlation, BXDs with thinner LV walls because of LV chamber dilation had higher Cu content. On the other hand, the BXD strains with lower cardiac Cu content had thicker LV walls, suggesting a significant negative association between cardiac Cu concentrations and myocardial hypertrophy. Taken together, Cu content imbalance in the heart may be associated with an undesirable remodeling in cardiac structure and geometry. Furthermore, our genetic mapping identified six QTLs associated with cardiac Cu concentrations in the BXD family. These QTLs are located on Chr X, Chr 2, Chr 4, Chr 6, Chr 8, and Chr 10; the 1.5-LOD region across these six QTLs contained a total of 217 genes. The candidate genes were narrowed down by employing a stringent scoring system that identified 21 candidate Cu-content-modifier genes, suggesting that cardiac Cu concentration is a multigene-regulated phenotype.

Among the 21 candidate genes, two genes, *Prex1* on Chr 2 and *Irx3* on Chr 8, met all the scoring criteria and received a score of 10. The *Prex1* gene encodes a guanine nucleotide exchange factor for the RHO family of small GTP-binding proteins (RACs). PREX1 is expressed in multiple tissues in both mouse and human and is known to activate RAC1. The Framingham Heart Study 100K Project, which analyzed genome-wide SNP (Affymetrix 100K GeneChip) associations with systolic and diastolic blood pressure found a significant association of *PREX1* rs6063312 SNP with diastolic and systolic blood pressure and arterial stiffness phenotypes as well as with MEF2C, a regulator of cardiac morphogenesis (36). There is no direct evidence that *Prex1* regulates Cu metabolism or transportation. However, it has been found that Cu transporter ATP7A is regulated by the RAC1 GTPase pathway (37) and interacts with a RAC1-binding protein IQGAP1 (38). *Prex1* might be involved in Cu transportation through the activation of RAC1 and ATP7A. *IRX3*, a member of the Iroquois homeobox gene family, has been shown to play a critical role in the precise regulation of intercellular gap junction coupling by modulating the transcription of connexins, Cx40, and Cx43, necessary for normal ventricular conduction (39). Mutations in *IRX3* cause ventricular fibrillation, the main cause of sudden cardiac death in humans (40). *Irx3* KO (Irx3^–/–^) mice develop ventricular conduction system defects with a lack of gap junctional channels during the early postnatal period due to enriched transcripts targeted by Nkx2.5 and/or Tbx5 (41). IRX3 affects obesity (42), which is associated with higher Cu in serum and tissues (43). Thus, further studies are required for understanding how IRX3 regulates Cu content in the heart.

Scored as 9, *Fam83d* on Chr 2 is known to be involved in the positive regulation of cell cycle G1/S phase transition, protein localization to the mitotic spindle, and regulation of intracellular signal transduction. *Fam83d* is expressed in skeletal muscle and is significantly induced in response to denervation and neurogenic atrophy involving MAP kinase and AKT/PKB (protein kinase B) signaling (44). However, there are very few studies pertaining to this gene and it needs to be explored further in the context of cardiac Cu-related associations identified.

Among 6 genes that scored 7, *Ankrd6* encodes ankyrin repeat domain 6, also known as diversin, a ubiquitous protein highly expressed in the heart, brain, and spinal cord in humans is shown to be involved in neural development *via* suppressing canonical Wnt signaling (45). Genetic SNPs in *ANKRD6* were associated with muscle hypertrophy and strength response to physical activity (46). Genes, *Slc35a1* (Chr 4) and *Slc26a10* (Chr 10) encode transporter proteins. SLC35A1 protein transports activated sugars into the endoplasmic reticulum (ER) and/or Golgi apparatus and *SLC35A1* mutations have been associated with congenital disorder of glycosylation II and platelet life span (47, 48). SLC26A10 is an anion transporter and has been found to be related to cortical thickness in Alzheimer’s disease (49). Two genes, *Usb1* and *Lrig3*, have recently been studied as therapeutic targets in poikiloderma with neutropenia and prostate cancer, respectively (50, 51), while *Ica1* encodes an autoantigen involved in autoimmune insulin-dependent diabetes mellitus and primary Sjogren’s syndrome (52). Therefore, further studies are required to define the functions of these genes in cardiac Cu regulation and heart physiology.

Interestingly, *Mmp2* with a score of 6 encodes matrix metalloproteinase 2 (MMP2), a Zn-binding member of MMPs, that has numerous substrates in cardiomyocytes that contribute to cardiac ischemia-reperfusion injury, cardiac remodeling, and heart failure (53). A study with hypertension and cardiac hypertrophy found that increased cardiac MMP2 contributes to the transition of concentric to eccentric LV hypertrophy and cardiac dysfunction (54). MMP2 serum levels are higher in patients with Wilson’s disease compared to healthy controls (55), supporting our findings of the association of cardiac Cu content with *Mmp2* expression in BXDs. Interestingly, Cu repletion recovered MMP2 level and reduced cardiac fibrosis in a rat model of pressure overload-induced cardiac hypertrophy (56). It has been shown that Cu could also increase Zn concentration in the heart, along with upregulation of MMP2 (12, 57), indicating that the balanced Cu content and MMP2 are important for cardiac morphology and function. The other genes noteworthy to mention are *Ctsa* and *Armcx1. Ctsa* encodes Cathepsin A (CatA), a serine carboxypeptidase that degrades extracellular peptides in lysosomes (58). A Cu chaperone, extracellular SOD (EC-SOD) is a substrate of CatA and a significant correlation between CatA expression and Cu content in normal aorta wall has been reported (59). Moreover, cardiomyocyte-specific CatA overexpression reduced EC-SOD levels, resulting in oxidative stress, inflammation, ECM remodeling, and myocyte hypertrophy *in vivo* (58), whereas inhibition of CatA attenuated infarction-induced heart failure (60). Located on Chr X, *Armcx1* encodes armadillo repeat-containing X-linked protein 1 that is involved in mitochondrial trafficking in neurons (61). Recently, *Armcx1* has been identified as a hub gene enriched in patients with ST-segment elevation myocardial infarction (62). Although these genes received a score of 6, their perceived involvement in cardiac remodeling warrants further studies to uncover the underlying pathomechanisms of ischemic heart disease.

In summary, we identified candidate genes that modulate cardiac Cu content using a systems genetics strategy. One significant and five suggestive QTLs have been mapped on different chromosomes and 21 candidate genes have been identified. *Prex1* and *Irx3* are suggested as potential modifier genes of cardiac Cu content, while *Ctsa, Mmp2*, and *Armcx1* may closely be related to ischemic heart disease. Taken together, Cu content in the heart is a multigene-regulated phenotype and further experimental investigations of candidate genes identified in our study will undoubtedly uncover and expand our current understanding of the role of Cu in heart performance and pathogenesis of cardiac diseases.

## Data availability statement

The datasets presented in this study can be found in online repositories. The names of the repository/repositories and accession number(s) can be found below: https://genenetwork.org/; GN1028.

## Ethics statement

The animal study was reviewed and approved by The University of Tennessee Health Science Center Animal Care and Use Committee.

## Author contributions

WJZ and LL: conceptualization and designs. AB, QG, B-OO, FX, CT-R, WYZ, CC, LL, and WJZ: data collection and analysis. AB, QG, AS-D, BJ, DL, JT, EP, LL, and WJZ: writing and editing the manuscript. All authors read and approved the final manuscript.

## Footnotes

1
http://genenetwork.org/

2
https://github.com/genetics-statistics/GEMMA

3
https://useast.ensembl.org/

4
http://www.informatics.jax.org/

5
www.rgd.mcw.edu

6
http://www.mousephenotype.org/

7
www.ebi.ac.uk/gwas

8
http://geneontology.org/

9
https://www.wikipathways.org/

10
https://www.genome.jp/kegg/

## Supplementary material

Supplementary File 1 Echocardiographic parameter values.
